# Supplementary figures and images for: Characterizing Microglial Signaling Dynamics During Inflammation Using Single‐Cell Mass Cytometry
Source: Glia. 2025 Jan 8;73(5):1022–35. doi: 10.1002/glia.24670 (PMC11920681; doi:10.1002/glia.24670)

Figure S1

A

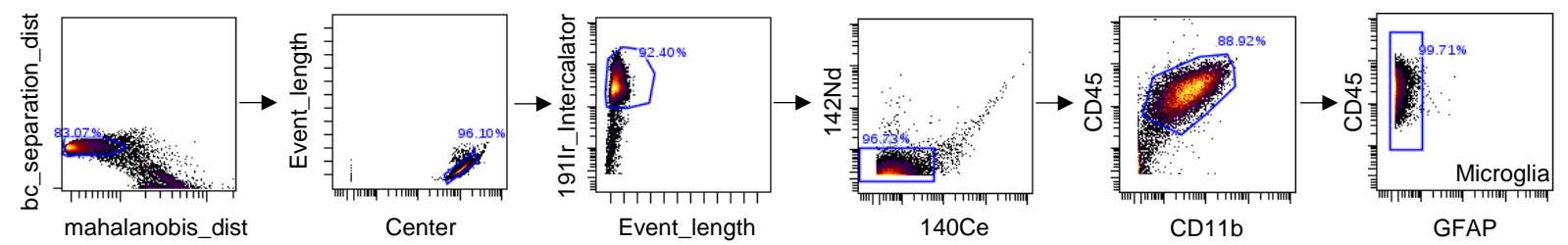

B

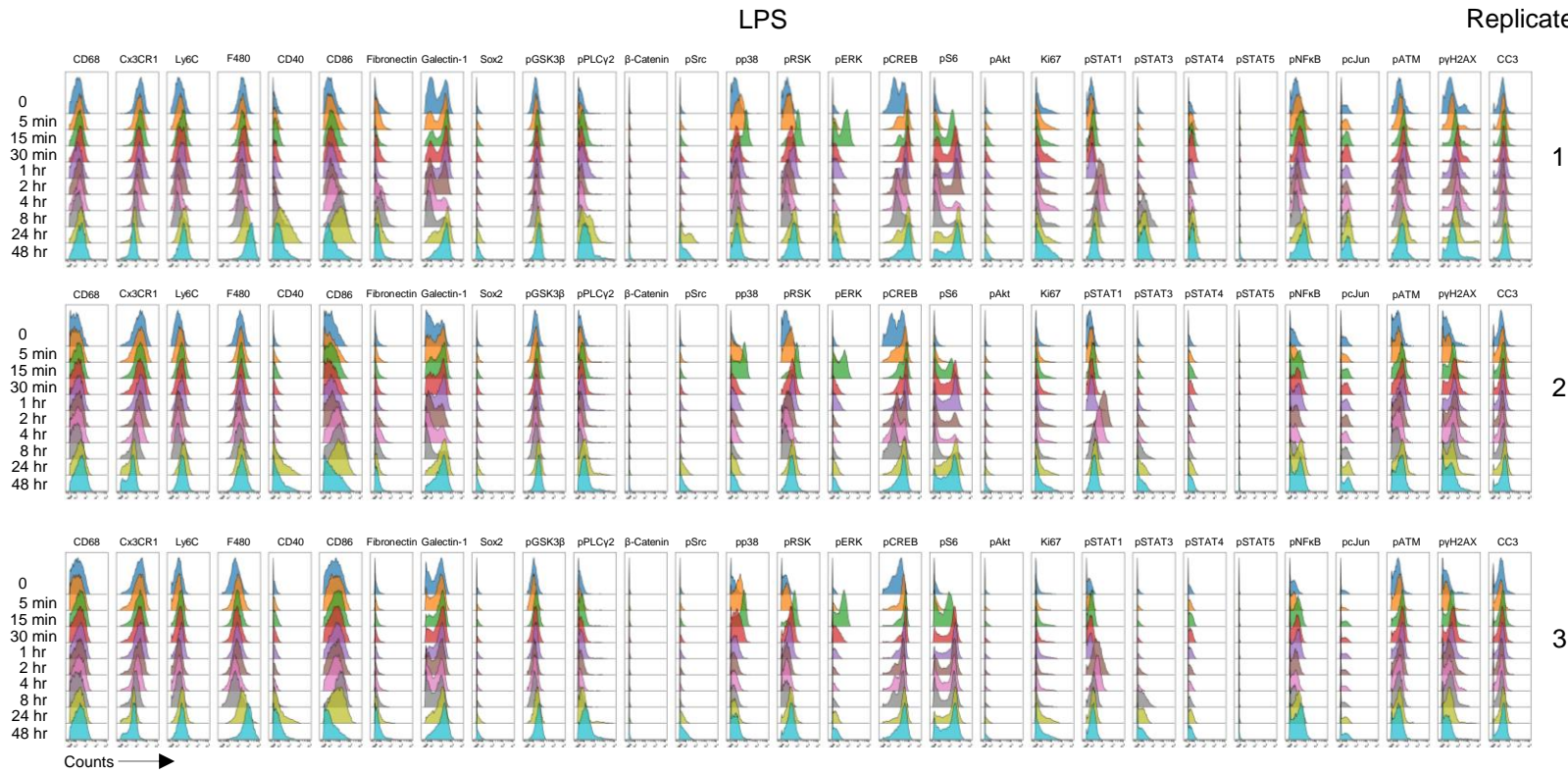

C

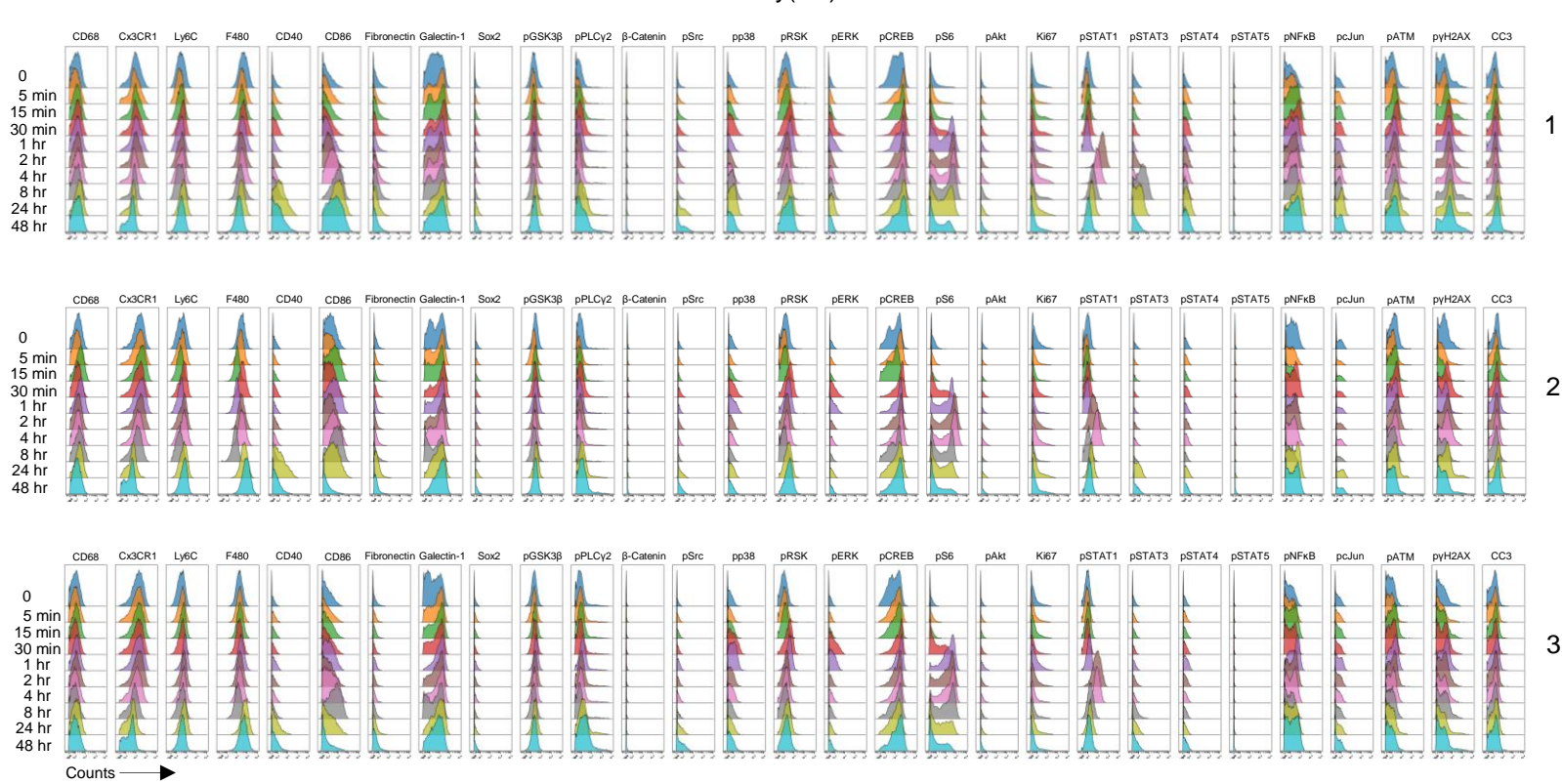

Supplement: Supplementary file 1 — Supplementary Figure 1 Replicate analysis of microglia‐only cultures treated with LPS or Poly(I:C). Gating strategy in Cytobank to isolate microglia. bc_separation_dist x mahalanobis_dist—gate to exclude non‐barcoded events. Event_length x Center—gate to exclude doublet events as well as events that fall outside the normal Gaussian discrimination parameters for mass cytometry. 191Ir_Intercalator x Event_length—gate to remove non‐cell events and dying/dead cells. 142Nd x 140Ce—gate to remove cerium contamination from normalization beads or environment. CD45 x CD11b—gate to select for microglia. CD45 x GFAP—secondary clean‐up gate to remove residual astrocyte contaminants (more relevant for mixed cultures but was also applied to pure cultures to maintain consistency) (A). Histograms depicting marker counts for all pure replicates in LPS and Poly(I:C) conditions (B). [file GLIA-73-1022-s008.pdf]

Figure S2

A

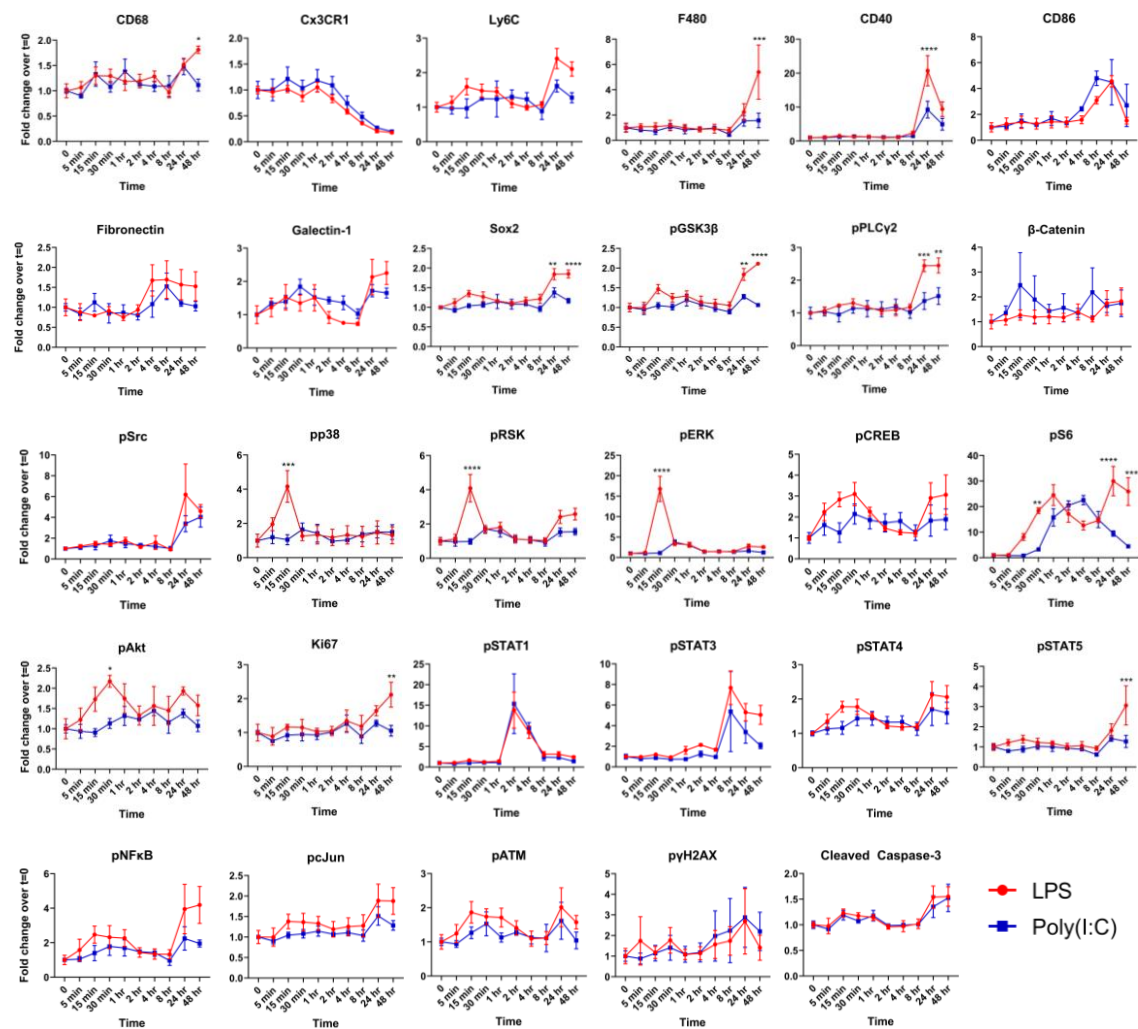

Supplement: Supplementary file 2 — Supplementary Figure 2 Line plots from LPS and Poly(I:C) treated microglial samples depicting signaling changes over time (A). Results are presented as fold changes (normalized to respective vehicle intensity). Data were analyzed by two‐way ANOVA followed by Šídák multiple comparisons test (A). Data are from at least three independent experiments and expressed as mean ± s.e.m. All data represent results taken from three independent cultures. Asterisks denote significance for LPS vs. Poly(I:C) responses at a given time point. *p < 0.05, **p < 0.01, ***p < 0.001, ****p < 0.0001. [file GLIA-73-1022-s002.pdf]

Figure S5

A

Microglia Cluster Heatmap

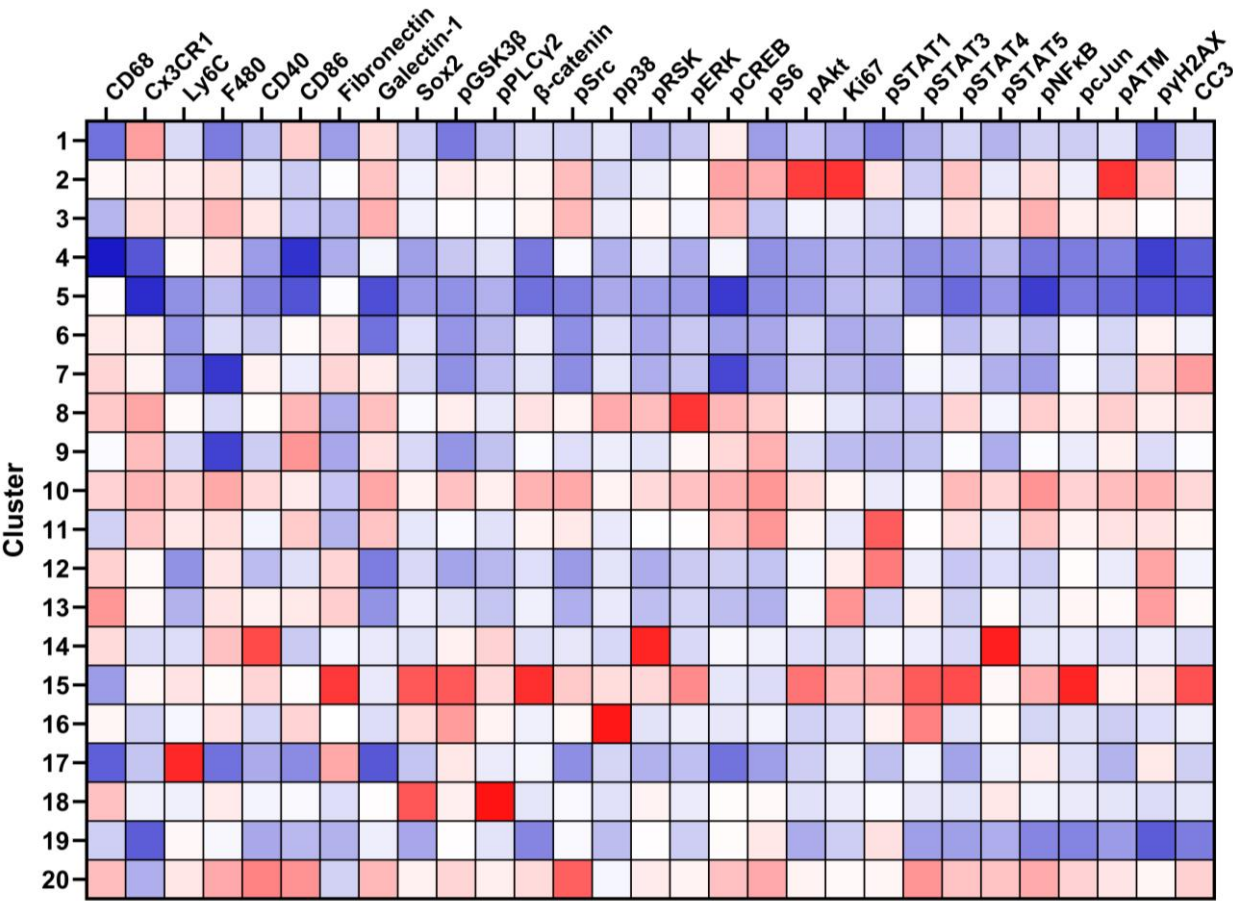

B

Microglia + Ast Cluster Heatmap

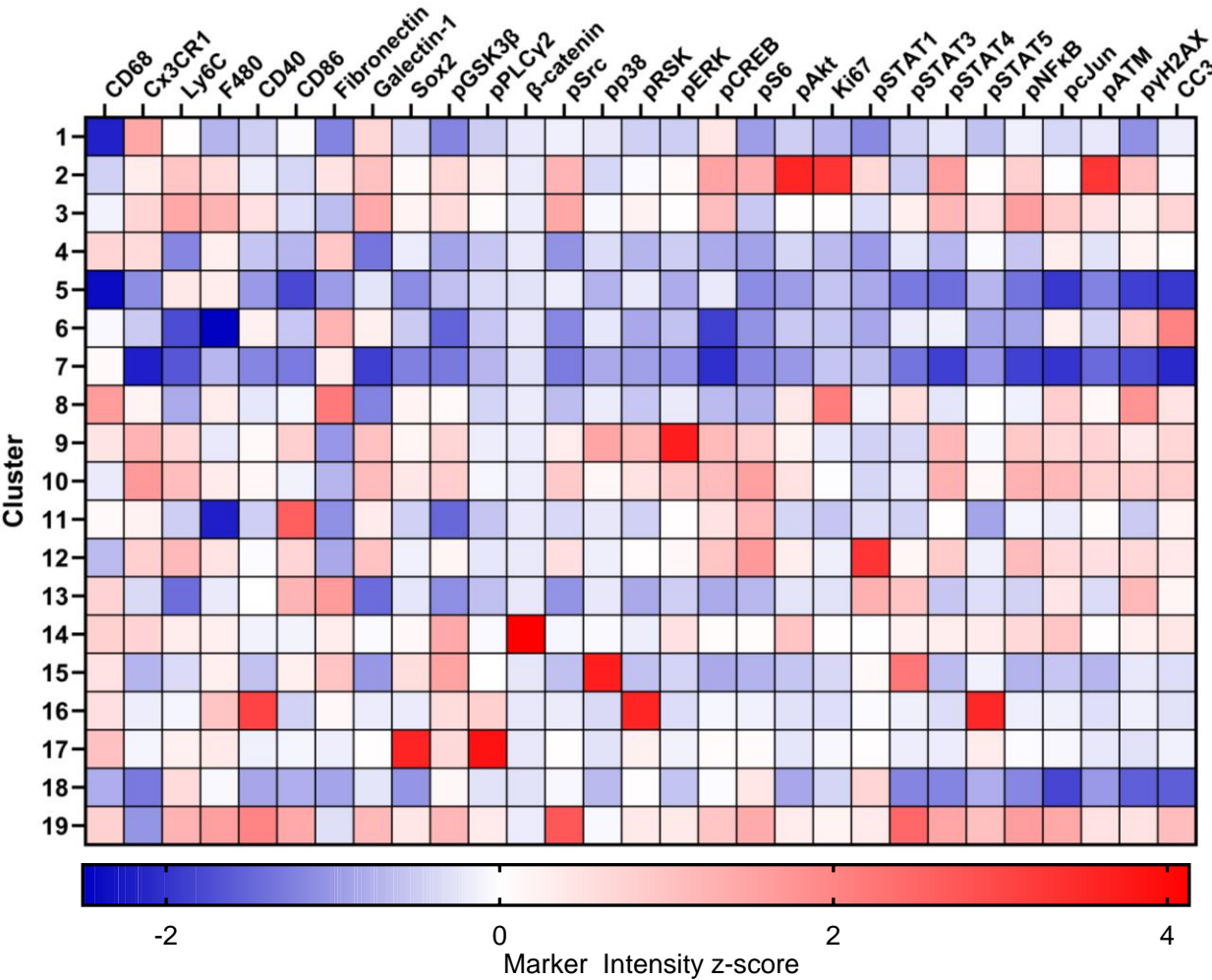

Supplement: Supplementary file 5 — Supplementary Figure 5 Z‐score heatmaps. To broadly highlight differences across clusters, z‐scores were calculated for each marker used for secondary clustering (clustering by signaling markers) across all identified clusters for microglia from microglia‐only cultures (A) or microglia from microglia‐astrocyte co‐cultures (B). Each cell is colored by relative marker expression with red representing higher expression (positive z‐scores) and blue representing lower expression (negative z‐scores) compared to the mean expression across all clusters. White indicates marker expression near the mean. [file GLIA-73-1022-s009.pdf]

Figure S6

A

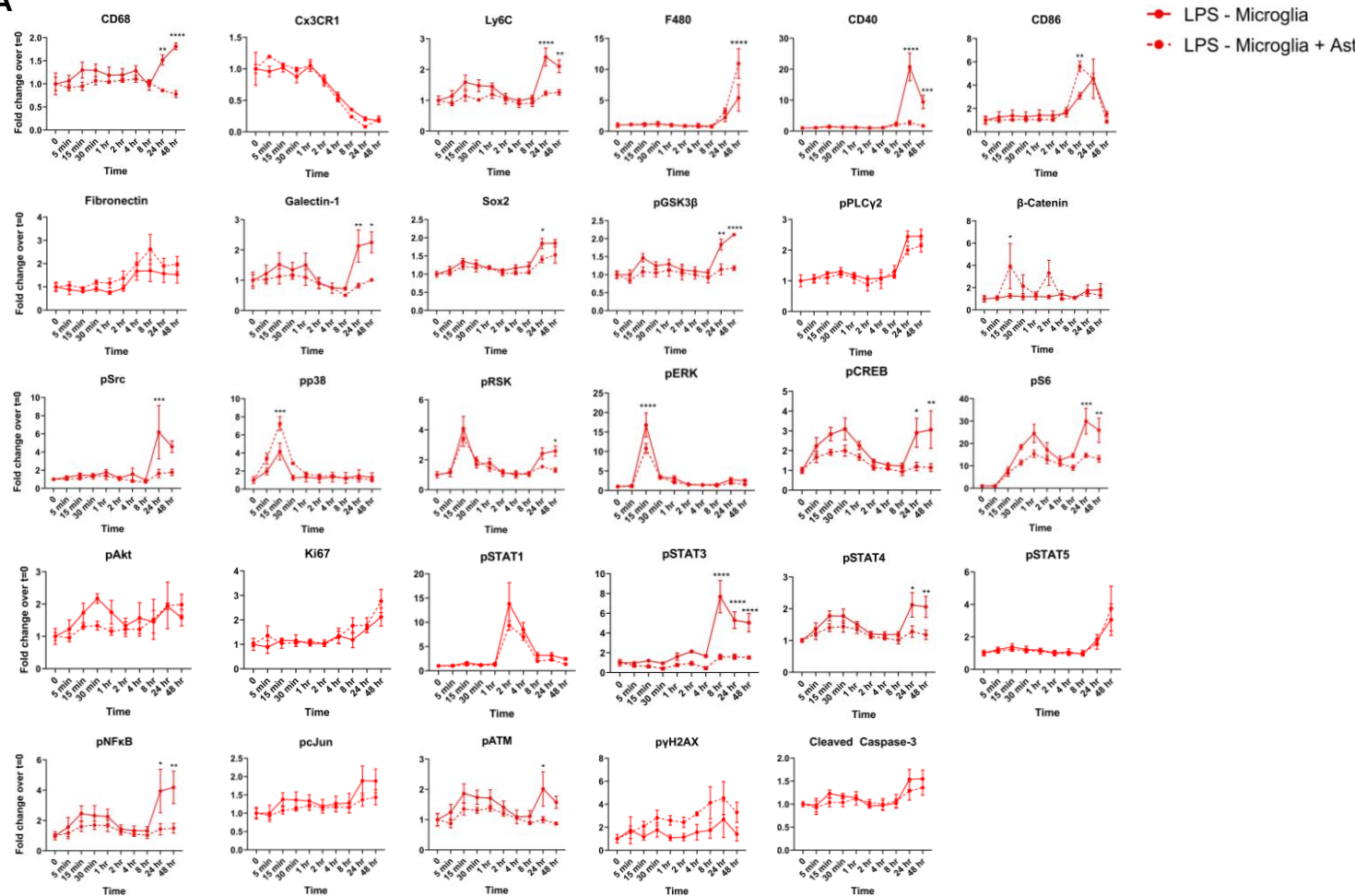

B

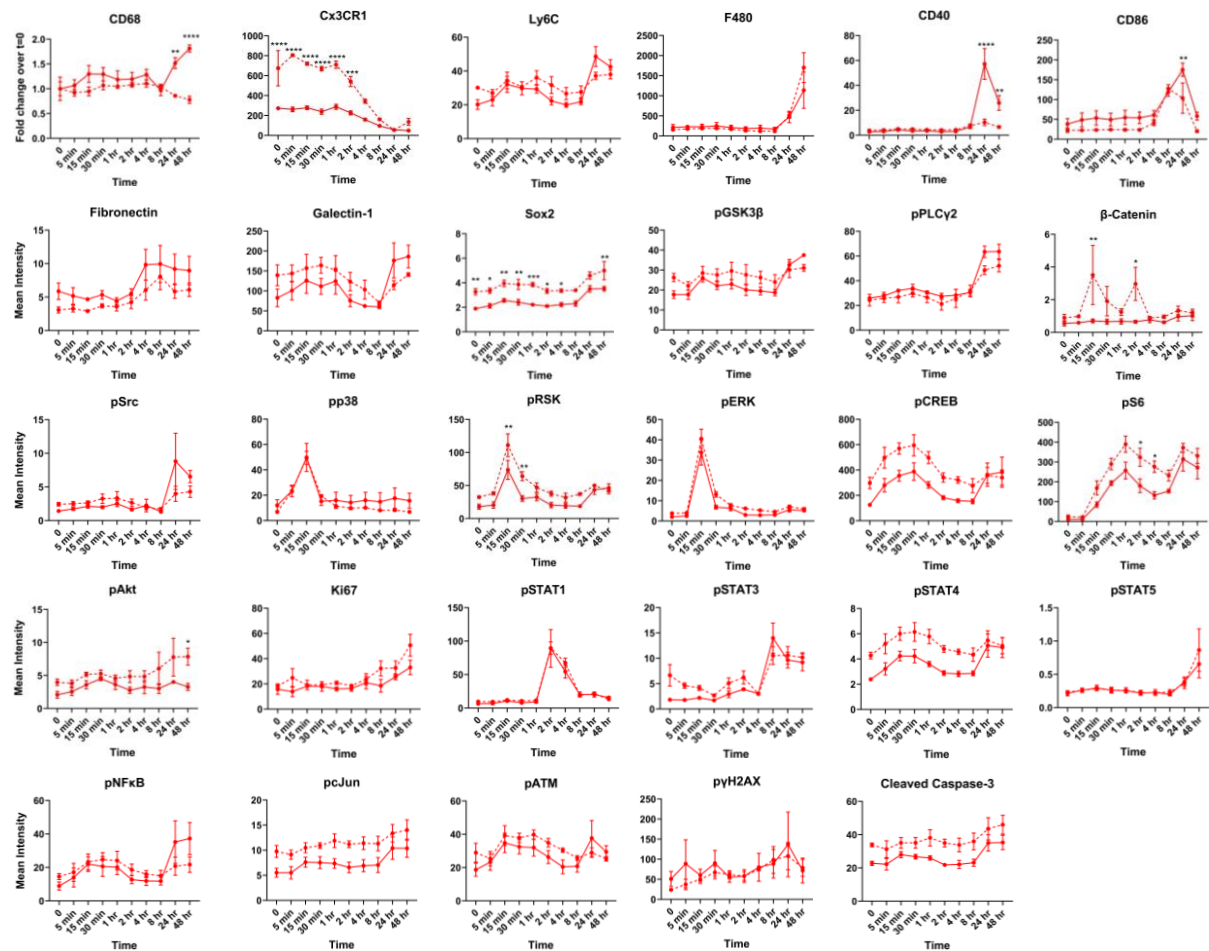

Supplement: Supplementary file 6 — Supplementary Figure 6 Summary of microglial LPS responses. Fold changes (A) or raw average intensities for all markers except CD11b, CD45, GFAP, and Olig2 (B) are shown for LPS‐treated microglia‐only and microglia+astrocyte cultures. Data were analyzed by two‐way ANOVA followed by Šídák multiple comparisons test (A, B). Data are from at least three independent experiments and expressed as mean ± s.e.m. All data represent results taken from three independent cultures. Asterisks denote significance for Microglia vs. Microglia + Ast responses at a given time point. *p < 0.05, **p < 0.01, ***p < 0.001, ****p < 0.0001. [file GLIA-73-1022-s011.pdf]

Figure S7

A

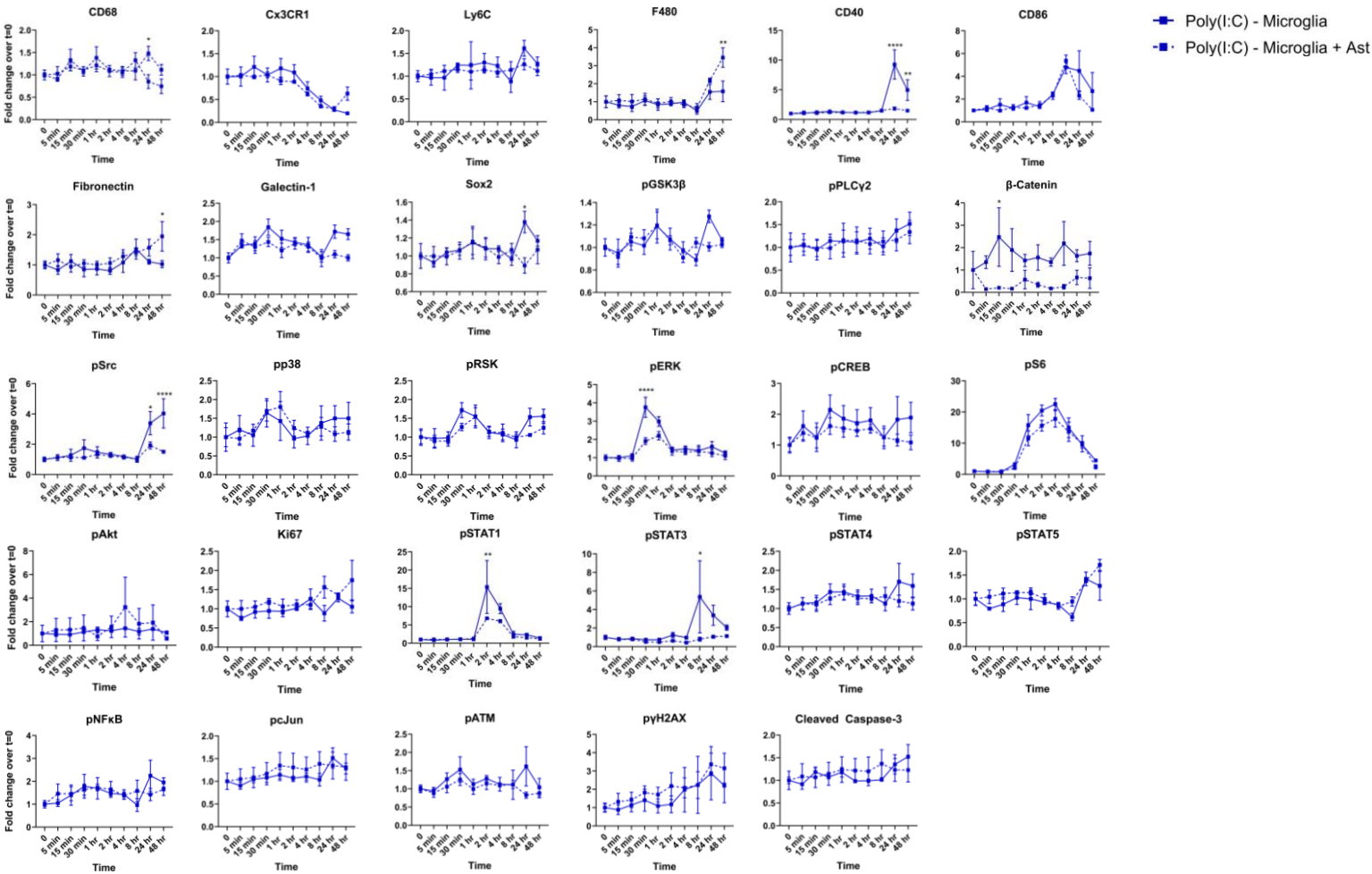

B

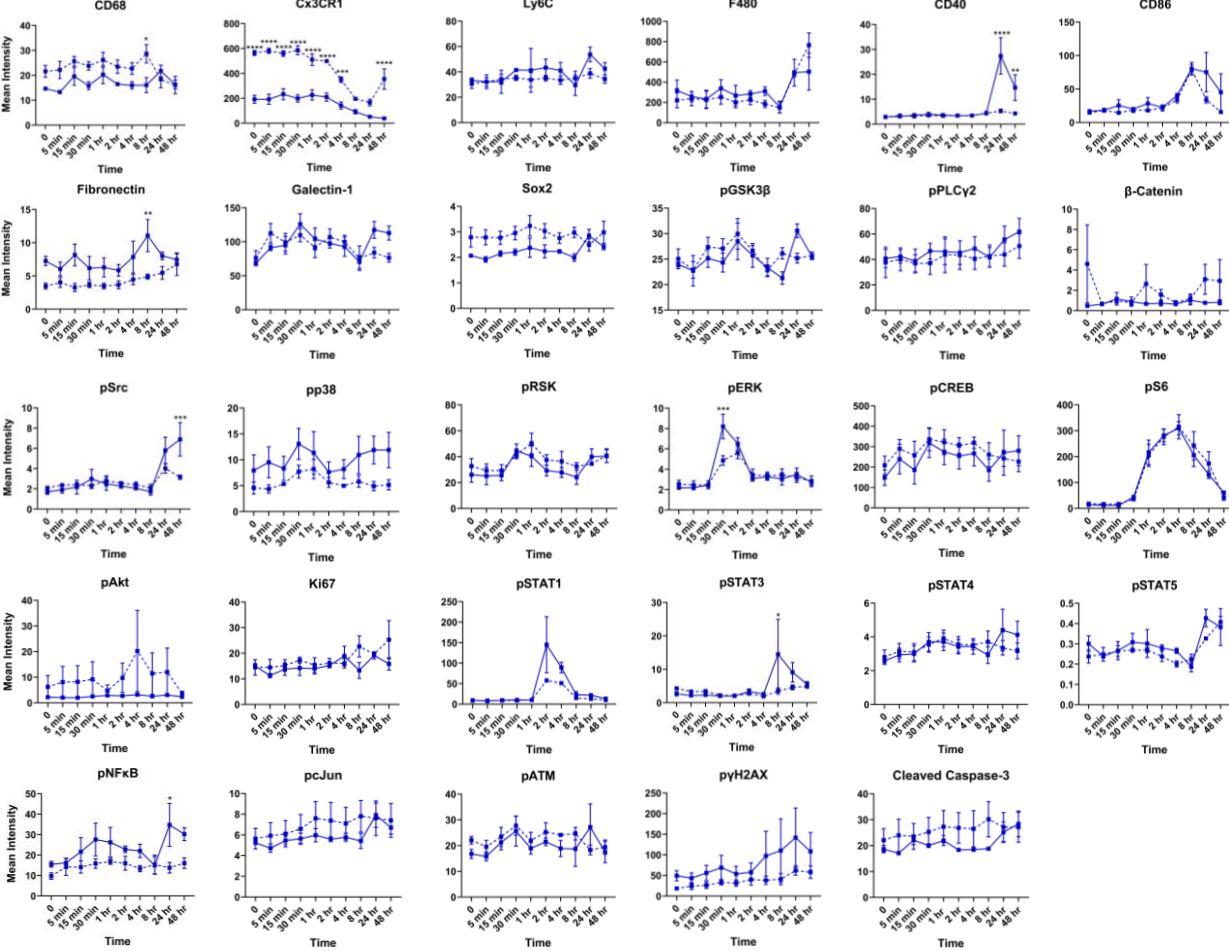

Supplement: Supplementary file 7 — Supplementary Figure 7 Summary of microglial Poly(I:C) responses. Fold changes (A) or raw average intensities for all markers except CD11b, CD45, GFAP, and Olig2 (B) are shown for Poly(I:C)‐treated microglia‐only and microglia+astrocyte cultures. Data were analyzed by two‐way ANOVA followed by Šídák multiple comparisons test (A, B). Data are from at least three independent experiments and expressed as mean ± s.e.m. All data represent results taken from three independent cultures. Asterisks denote significance for Microglia vs. Microglia + Ast responses at a given time point. *p < 0.05, **p < 0.01, ***p < 0.001, ****p < 0.0001. [file GLIA-73-1022-s003.pdf]

Figure S8

A

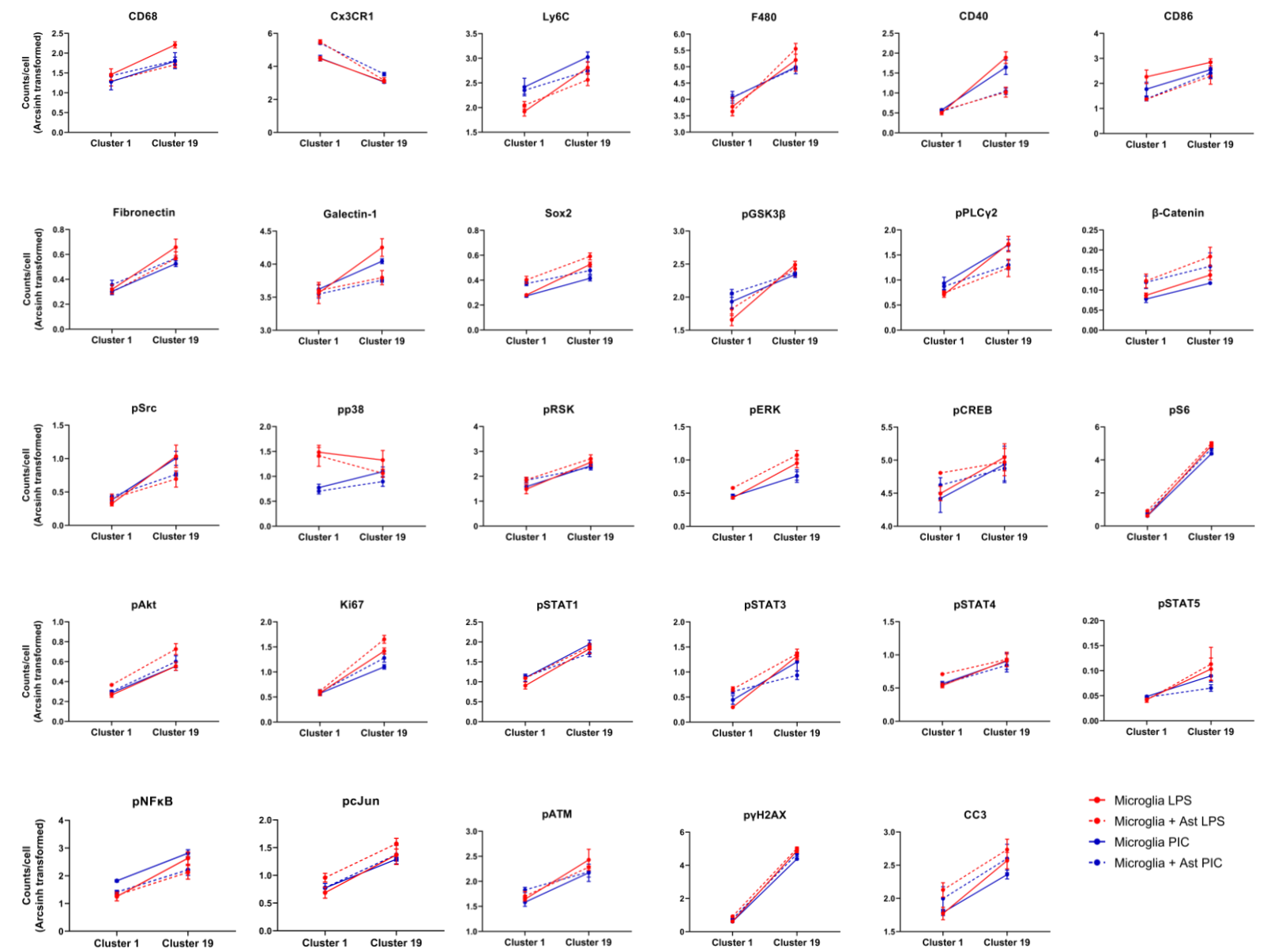

Supplement: Supplementary file 8 — Supplementary Figure 8 Comparison of Cluster 1 vs. Cluster 19 marker intensity. Counts/cell for all markers used in secondary clustering are shown for Cluster 1 and Cluster 19 for all treatment conditions (A). [file GLIA-73-1022-s013.pdf]

Figure S9

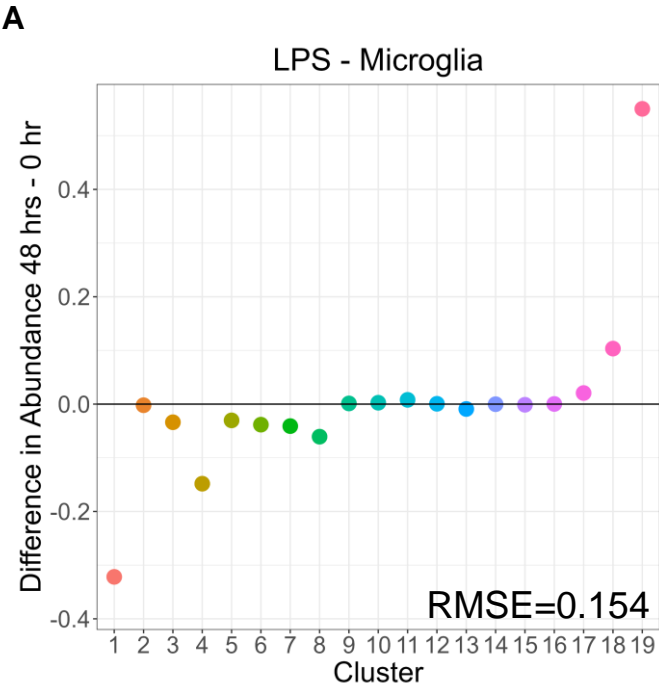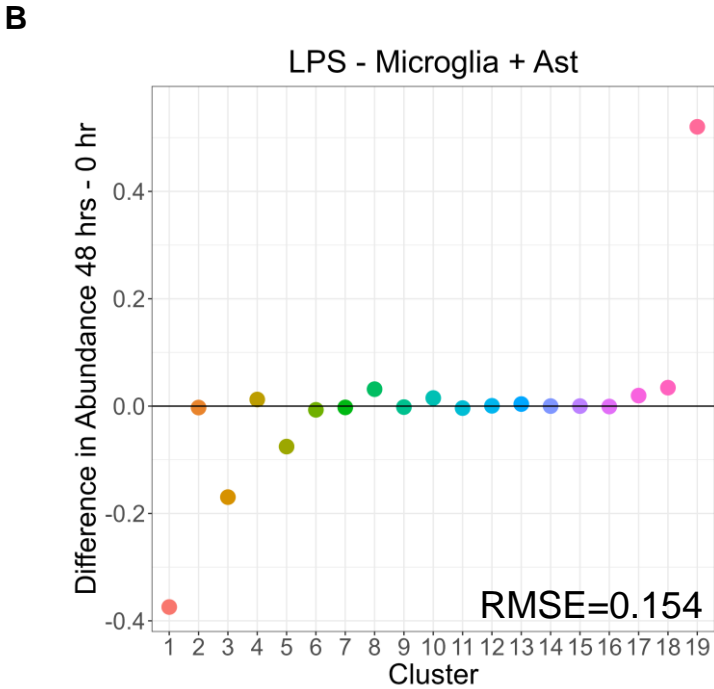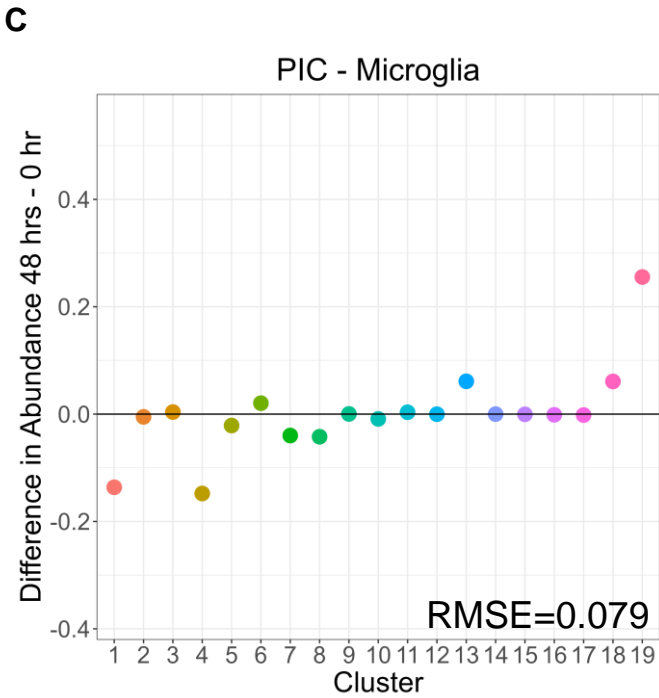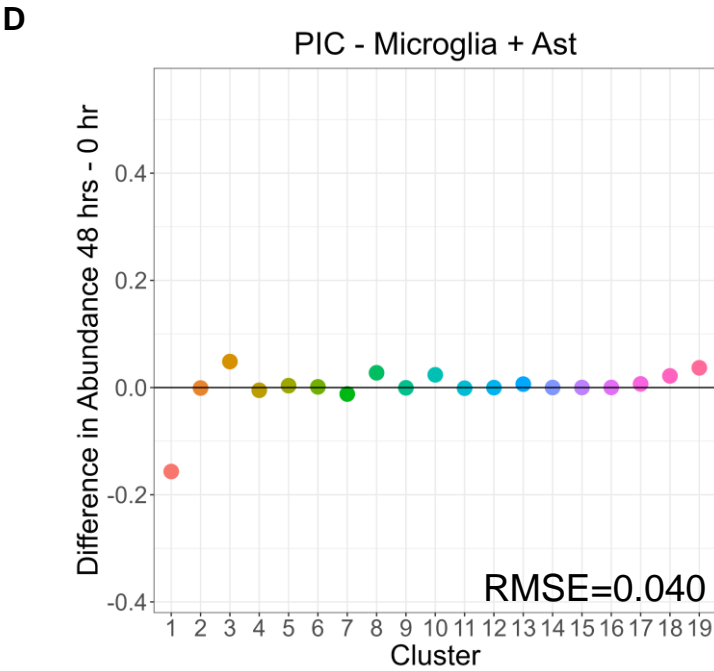

Supplement: Supplementary file 9 — Supplementary Figure 9 Microglia from microglia + astrocyte Poly(I:C) setting show the greatest return to baseline after 48 h. Differences in cluster abundance between the 48 h and 0 h mark were calculated for microglia‐only (LPS) (A), microglia + astrocyte (LPS) (B), microglia‐only (Poly(I:C)) (C), and microglia + astrocyte (Poly(I:C)) conditions (D). Root‐mean squared errors (using 0 as the expected value) are reported on lower‐right. [file GLIA-73-1022-s007.pdf]

Figure S10

A

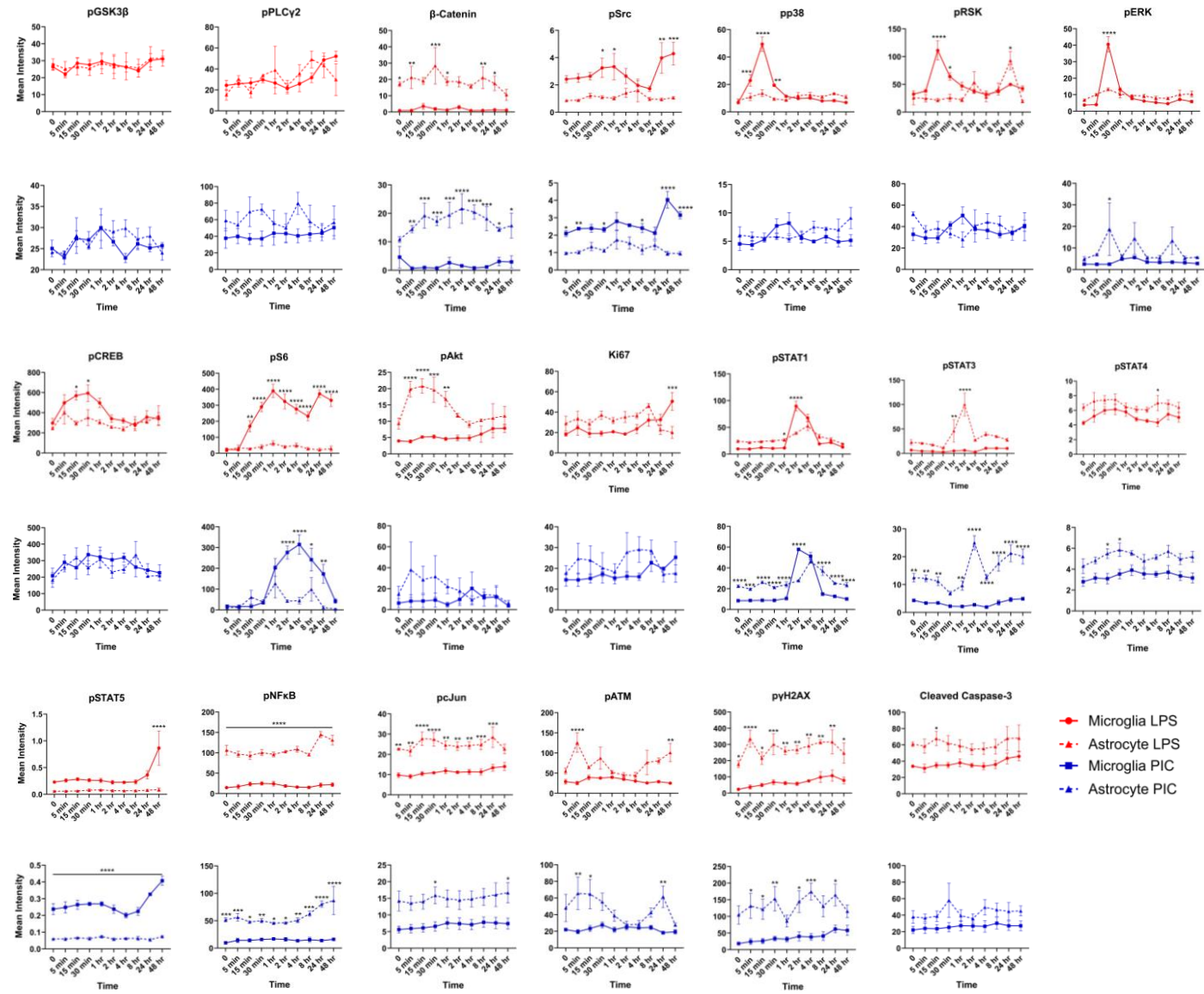

Supplement: Supplementary file 10 — Supplementary Figure 10 Mean marker intensity line plots for astrocytes from LPS or Poly(I:C) time courses. Microglial responses are also included for comparison (A). Data were analyzed by two‐way ANOVA followed by Šídák multiple comparisons test (B). Data are from at least three independent experiments and expressed as mean ± s.e.m. All data represent results taken from three independent cultures. Asterisks denote significance for Microglia vs. Astrocyte mean intensities at a given time point. *p < 0.05, **p < 0.01, ***p < 0.001, ****p < 0.0001. [file GLIA-73-1022-s012.pdf]
