# Supplementary material for: Characterizing Microglial Signaling Dynamics During Inflammation Using Single‐Cell Mass Cytometry
Source: Glia. 2025 Jan 8;73(5):1022–35. doi: 10.1002/glia.24670 (PMC11920681; doi:10.1002/glia.24670)

Figure S3

A Uncropped gels corresponding to Figure 3C

Order for all blots:

Ladder – Vehicle – 5 min LPS – 15 min LPS – 1 hr LPS – 2 hr LPS – 4 hr LPS – 24 hr LPS – Empty - Ladder

pp38

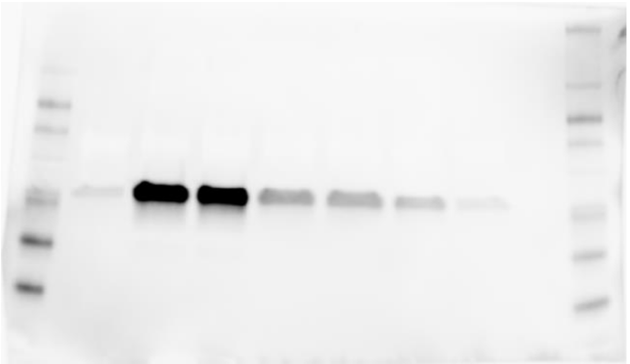

p38

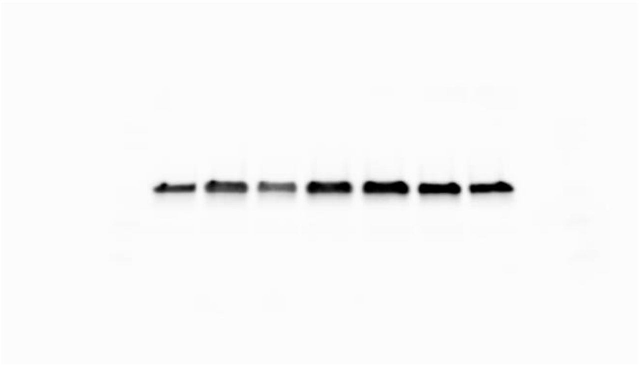

pERK

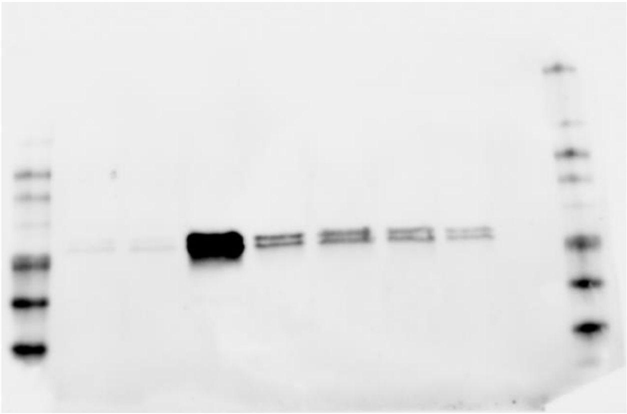

ERK

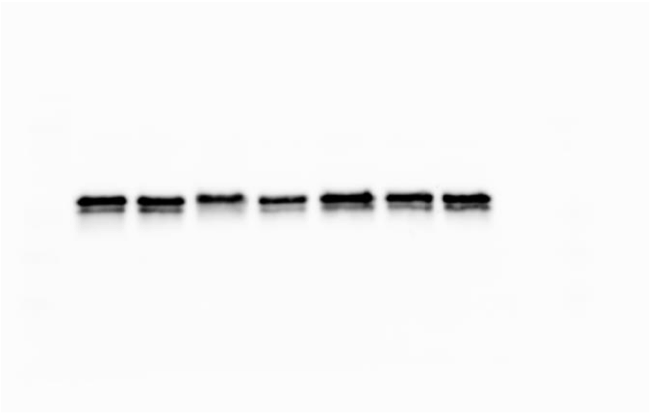

pSTAT1

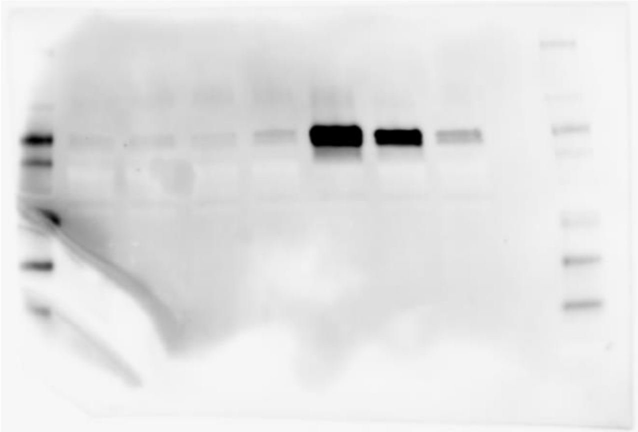

STAT1

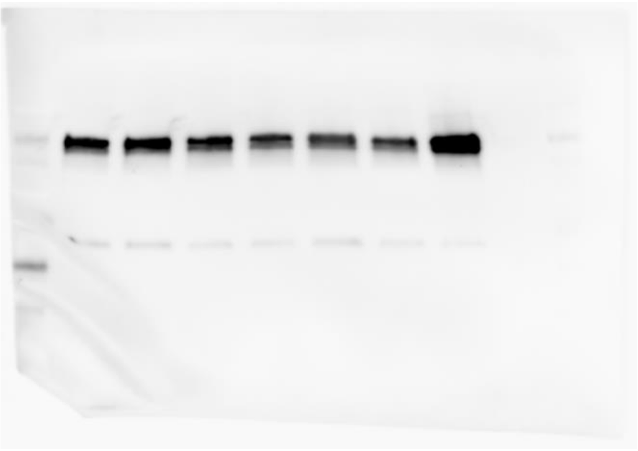

Supplement: Supplementary file 3 — Supplementary Figure 3 Uncropped gels for Figure 2C. [file GLIA-73-1022-s006.pdf]
