# Supplementary material for: Characterizing Microglial Signaling Dynamics During Inflammation Using Single‐Cell Mass Cytometry
Source: Glia. 2025 Jan 8;73(5):1022–35. doi: 10.1002/glia.24670 (PMC11920681; doi:10.1002/glia.24670)
Supplement: Supplementary file 4 — Supplementary Figure 4 Isolating microglia for high‐dimensional analysis. Cell events from microglia‐only samples were exported from Cytobank and clustered on identity markers (A). Violin plot showing protein expression from clusters in (A). Clusters 7,11, and 15 were deemed to be non‐microglia and excluded from secondary clustering (B). [file GLIA-73-1022-s004.pdf]

Figure S4

A

1. Initial clustering on identity markers
2. Isolate microglia (CD11b^{hi}, CD45^{hi}, GFAP^{lo}, and Olig2^{lo})
3. Recluster microglia on all markers except CD11b, CD45, GFAP, and Olig2

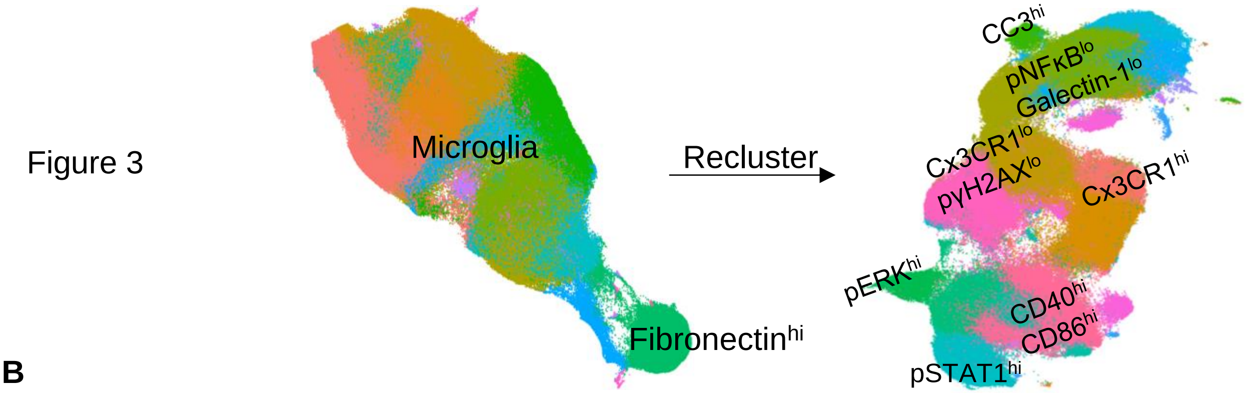

B

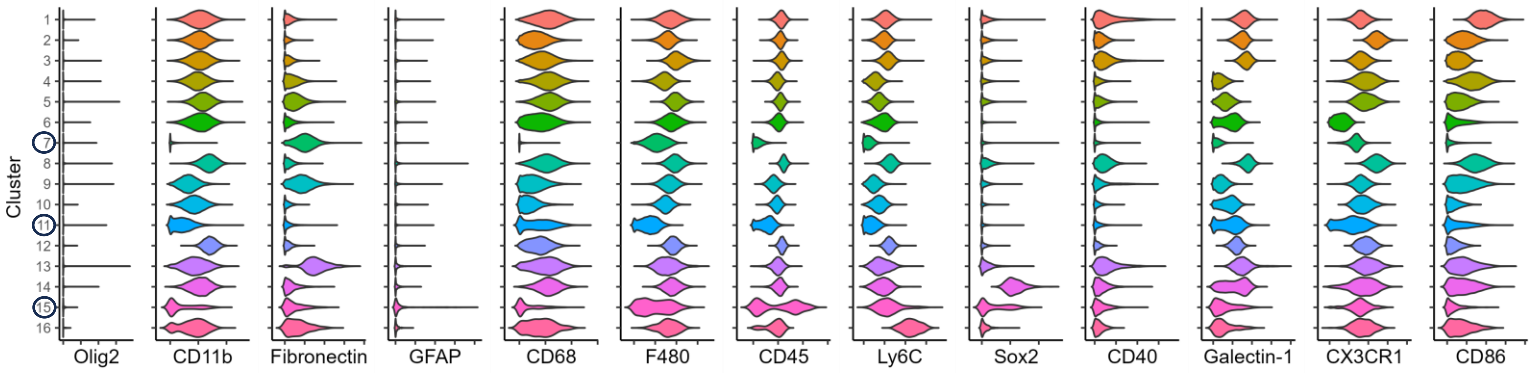

C

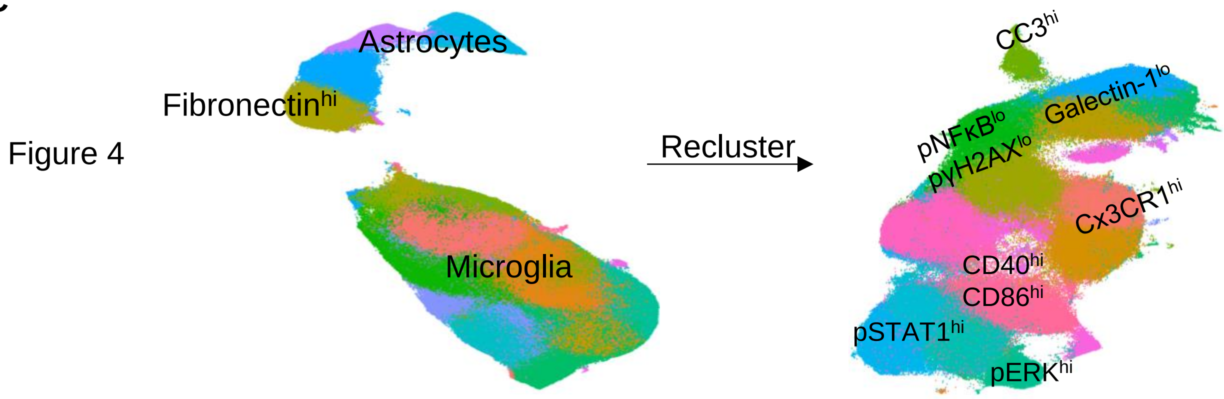

D

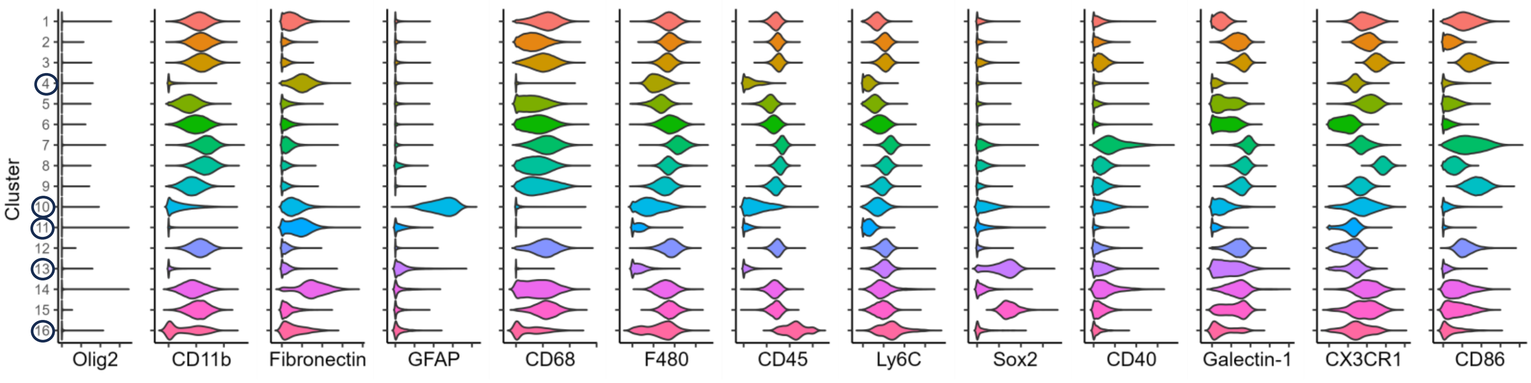

○ Denotes non-microglial clusters that were excluded for secondary round of clustering
