# Supplementary material for: Characterizing Microglial Signaling Dynamics During Inflammation Using Single‐Cell Mass Cytometry
Source: Glia. 2025 Jan 8;73(5):1022–35. doi: 10.1002/glia.24670 (PMC11920681; doi:10.1002/glia.24670)
Supplement: Supplementary file 11 — Supplementary Table 1 Mass cytometry antibody panel. Complete list of all markers used, concentrations of antibodies, and vendor information for mass cytometry studies. [file GLIA-73-1022-s005.pdf]

Table S1

A

Mass Cytometry Antibody Panel

Metal	Antigen	Concentration (ng/mL)	Vendor Information
In113	Olig2	30	Millipore (clone 211F1.1)
Cd114	Cd11b	55	Biolegend (M1/70)
In115	Fibronectin	150	BD Biosciences (610078)
La139	Phospho-GSK3β (S9)	1000	CST (D85E12)
Pr141	GFAP	25	BD Biosciences (556330)
Nd143	CD68	25	Biolegend (FA-11)
Nd144	Phospho-PLCγ2 (Y759)	2x	Fluidigm (K86-689.37)
Nd145	Phospho-RSK (S320)	2000	R&D Systems (AF3369)
Nd146	F4/80	1x	Fluidigm (BM8)
Sm147	Phospho-STAT5 (Y694)	1x	Fluidigm (47)
Sm149	CD45	30	Biolegend (30-F11)
Nd150	Ly6C	1x	Biolegend (HK1.4)
Sm152	Ki67	2000	Fluidigm (B56)
Eu153	Phospho-STAT1 (Y701)	1x	Fluidigm (58D6)
Sm154	Phospho-Src (Y418)	600	BD Biosciences (K98-37)
Gd156	Phospho-p38 (T180/Y182)	1x	Fluidigm (D3F9)
Gd158	Phospho-STAT3 (Y705)	1x	Fluidigm (4/P-STAT3)
Tb159	Phospho-ATM (S1981)	700	Biolegend (10H11.E12)
Gd160	Sox2	1000	R&D Systems (245610)
Dy161	CD40	1x	Fluidigm (HM40-3)
Dy162	Galectin-1	300	R&D Systems (AF1245)
Dy164	Cx3CR1	1x	Fluidigm (SA011F11)
Ho165	Phospho-γH2AX (S139)	700	Biolegend (2F3)
Er166	Phospho-NFκB (S529)	1x	Fluidigm (K10x)
Er168	Phospho-Akt (S473)	2000	BD Biosciences (560397)
Tm169	Phospho-cJun (S73)	5000	CST (D47G9)
Er170	βCatenin	50	CST (6B3)
Yb171	Phospho-p44/42 MAPK (ERK1/2) (T202/Y204)	1/3x	CST (D13.14.4E)
Yb172	CD86	1x	Fluidigm (GL1)
Yb173	CC3	5000	BD Biosciences (C92-605)
Yb174	Phospho-STAT4 (Y693)	1/3x	Fluidigm
Lu175	Phospho-S6 (S235/S236)	500	Fluidigm (N7-548)
Yb176	Phospho-Creb (S133)	1/3x	Fluidigm (87G3)
