# Supplementary material for: Characterizing Microglial Signaling Dynamics During Inflammation Using Single‐Cell Mass Cytometry
Source: Glia. 2025 Jan 8;73(5):1022–35. doi: 10.1002/glia.24670 (PMC11920681; doi:10.1002/glia.24670)
Supplement: Supplementary file 13 — Supplementary Table 3 Percentage breakdowns of identified cell types in cultures used for all experiments. % Other represents Fibronectinhi cells that were low in expression for other identity markers. Percentages in black reference microglia‐only cultures while percentages in red reference microglia + astrocyte co‐cultures. [file GLIA-73-1022-s010.pdf]

Table S3

LPS (Micro/Micro + Ast)			
Time	% Microglia	% Astrocyte	% Other
0	Rep 1: 86.1/67.1<br>Rep 2: 71.0/65.9<br>Rep 3: 75.8/62.0	Rep 1: 1.8/29.7<br>Rep 2: 4.1/28.7<br>Rep 3: 9.2/34.6	Rep 1: 12.0/3.2<br>Rep 2: 24.8/5.4<br>Rep 3: 15.0/3.4
5 min	Rep 1: 90.0/64.4<br>Rep 2: 83.9/67.6<br>Rep 3: 85.1/69.0	Rep 1: 1.3/32.5<br>Rep 2: 2.8/25.7<br>Rep 3: 4.8/28.4	Rep 1: 8.7/3.1<br>Rep 2: 13.3/6.7<br>Rep 3: 10.1/2.6
15 min	Rep 1: 91.7/69.8<br>Rep 2: 89.0/70.2<br>Rep 3: 93.2/71.5	Rep 1: 1.4/27.8<br>Rep 2: 1.2/23.7<br>Rep 3: 0.2/26.5	Rep 1: 6.9/2.3<br>Rep 2: 9.7/6.0<br>Rep 3: 6.6/2.0
30 min	Rep 1: 92.2/59.8<br>Rep 2: 86.4/70.4<br>Rep 3: 88.2/70.9	Rep 1: 1.1/35.9<br>Rep 2: 1.7/24.3<br>Rep 3: 3.9/26.3	Rep 1: 6.6/4.3<br>Rep 2: 11.9/5.3<br>Rep 3: 7.9/2.8
1 hr	Rep 1: 90.8/62.2<br>Rep 2: 85.4/61.7<br>Rep 3: 88.8/72.6	Rep 1: 1.6/34.1<br>Rep 2: 2.2/30.6<br>Rep 3: 3.8/25.0	Rep 1: 7.6/3.7<br>Rep 2: 12.4/7.7<br>Rep 3: 7.3/2.4
2 hr	Rep 1: 83.2/50.9<br>Rep 2: 73.6/56.5<br>Rep 3: 76.7/55.3	Rep 1: 2.5/42.3<br>Rep 2: 3.6/32.8<br>Rep 3: 8.0/39.9	Rep 1: 14.3/6.8<br>Rep 2: 22.7/10.7<br>Rep 3: 15.3/4.8
4 hr	Rep 1: 79.9/49.2<br>Rep 2: 71.7/43.5<br>Rep 3: 72.3/49.4	Rep 1: 1.8/42.6<br>Rep 2: 4.9/36.9<br>Rep 3: 8.2/42.3	Rep 1: 18.3/8.2<br>Rep 2: 23.3/19.6<br>Rep 3: 19.5/8.3
8 hr	Rep 1: 77.4/31.3<br>Rep 2: 84.6/32.4<br>Rep 3: 79.2/51.8	Rep 1: 2.2/53.4<br>Rep 2: 3.3/54.9<br>Rep 3: 6.2/40.3	Rep 1: 20.4/15.3<br>Rep 2: 12.1/12.7<br>Rep 3: 14.6/7.9
24 hr	Rep 1: 95.0/48.6<br>Rep 2: 96.8/59.0<br>Rep 3: 95.1/54.5	Rep 1: 0.6/43.4<br>Rep 2: 0.9/30.0<br>Rep 3: 2.3/37.9	Rep 1: 4.4/7.9<br>Rep 2: 2.3/11.0<br>Rep 3: 2.6/7.6
48 hr	Rep 1: 95.7/46.9<br>Rep 2: 96.7/53.6<br>Rep 3: 94.7/64.2	Rep 1: 1.3/41.7<br>Rep 2: 1.5/33.3<br>Rep 3: 2.8/29.2	Rep 1: 3.0/11.4<br>Rep 2: 1.8/13.2<br>Rep 3: 2.5/6.6

Poly(I:C) (Micro/Micro + Ast)			
Time	% Microglia	% Astrocyte	% Other
0	Rep 1: 90.1/57.0<br>Rep 2: 83.8/60.9<br>Rep 3: 89.9/53.2	Rep 1: 1.2/37.4<br>Rep 2: 2.5/30.1<br>Rep 3: 2.2/42.6	Rep 1: 8.8/5.5<br>Rep 2: 13.6/9.1<br>Rep 3: 7.9/4.2
5 min	Rep 1: 88.5/52.9<br>Rep 2: 91.0/64.0<br>Rep 3: 94.0/59.5	Rep 1: 1.6/39.7<br>Rep 2: 1.8/28.3<br>Rep 3: 1.7/36.9	Rep 1: 9.9/7.4<br>Rep 2: 7.1/7.6<br>Rep 3: 4.3/3.6
15 min	Rep 1: 89.1/57.0<br>Rep 2: 87.4/66.0<br>Rep 3: 95.1/57.8	Rep 1: 0.9/36.2<br>Rep 2: 4.5/29.1<br>Rep 3: 1.3/39.3	Rep 1: 10.0/6.9<br>Rep 2: 8.1/4.9<br>Rep 3: 3.6/2.9
30 min	Rep 1: 91.1/57.1<br>Rep 2: 94.8/64.6<br>Rep 3: 95.3/55.0	Rep 1: 0.7/36.8<br>Rep 2: 0.8/28.9<br>Rep 3: 1.8/41.1	Rep 1: 8.2/6.1<br>Rep 2: 4.4/6.5<br>Rep 3: 2.9/3.9
1 hr	Rep 1: 89.1/51.0<br>Rep 2: 90.1/63.7<br>Rep 3: 97.8/58.3	Rep 1: 0.9/40.9<br>Rep 2: 4.4/32.7<br>Rep 3: 1.1/40.1	Rep 1: 10.0/8.1<br>Rep 2: 5.5/3.6<br>Rep 3: 1.1/1.6
2 hr	Rep 1: 89.7/51.1<br>Rep 2: 91.2/67.7<br>Rep 3: 95.5/53.5	Rep 1: 0.9/41.5<br>Rep 2: 1.2/26.5<br>Rep 3: 1.5/42.9	Rep 1: 9.3/7.4<br>Rep 2: 7.6/5.7<br>Rep 3: 3.1/3.6
4 hr	Rep 1: 84.1/47.4<br>Rep 2: 92.2/66.4<br>Rep 3: 96.6/53.7	Rep 1: 1.1/42.7<br>Rep 2: 1.0/26.0<br>Rep 3: 1.3/42.5	Rep 1: 14.9/9.9<br>Rep 2: 6.7/7.6<br>Rep 3: 2.1/3.9
8 hr	Rep 1: 77.9/46.5<br>Rep 2: 81.4/43.5<br>Rep 3: 93.7/52.3	Rep 1: 1.6/42.2<br>Rep 2: 6.2/40.8<br>Rep 3: 1.6/42.6	Rep 1: 20.4/11.3<br>Rep 2: 12.4/15.7<br>Rep 3: 4.7/5.1
24 hr	Rep 1: 93.4/52.1<br>Rep 2: 93.9/61.0<br>Rep 3: 96.1/36.3	Rep 1: 1.0/36.9<br>Rep 2: 0.6/29.5<br>Rep 3: 0.8/53.7	Rep 1: 5.5/11.0<br>Rep 2: 5.5/9.4<br>Rep 3: 3.1/10.0
48 hr	Rep 1: 91.1/52.9<br>Rep 2: 96.0/56.7<br>Rep 3: 95.7/28.6	Rep 1: 3.1/35.6<br>Rep 2: 0.7/32.6<br>Rep 3: 1.0/55.7	Rep 1: 5.8/11.5<br>Rep 2: 3.3/10.7<br>Rep 3: 3.4/15.8
